# Cold Plague, or Malignant Bilious Pneumonia

**Published:** 1847-05

**Authors:** James D. Rivers

**Affiliations:** Hall County, Ga.


MEDICAL EXAMINER

AND

RECORD OF MEDICAL SCIENCE.

NEW SERIES.—No. XXIX.—MAY, 1847.

### ORIGINAL COMMUNICATIONS.

*Cold Plague, or Malignant Bilious Pneumonia.* By JAMES D. RIVERS, M. D., of Hall County, Ga.

The February number of the Examiner contains an article extracted from the Southern Medical and Surgical Journal, in which the writer makes some remarks in reference to a disease "which has prevailed more extensively in Georgia and South Carolina (and perhaps in other Southern states) during the last year or two than formerly, and which has been attended with an extraordinary degree of mortality." The disease to which the writer alludes, under the name of "Pneumonia," or "Pleuro Pneumonia," is well known to the people of North Carolina, South Carolina and Georgia, as the "Cold Plague;" and from the little experience that I have had in some fifteen or twenty cases, I am induced to believe that this latter appellation, or the more scientific one of Malignant Bilious Pneumonia, is preferable to the one which the writer has given, and is more descriptive of the malady than any other name that has come to my notice. The disease called the "Black Tongue," which prevailed to such an alarming extent in the north-western section of this State last year, is, I suspect, another form of the Cold Plague. With these remarks by way of prelude, I propose to make a few observations in reference to the general characters of the disease as developed in the cases that have fallen under my charge, and the method of treatment adopted, and then to give a detailed account of a few of the most important cases which I have had; merely remarking beforehand, that all my patients affected with this disorder, to the number of about twenty, recovered under the prescribed treatment.

The diagnostic symptoms of the Malignant Bilious Pneumonia of this section of country, are rigors, but never a shaking chill; coldness of the body and extremities, pain in the forehead, generally an inch or two above the orbits of one of the eyes, most commonly over the left eye, severe pain in the chest, usually on the right side, pain in the region of the liver and pain in the abdomen. As the malady advances in its inflammatory course, the tongue thickens and expands laterally. In the outset of the disorder the pain is metastatic, sometimes moving from the head to either lung, usually the right one, frequently to the superior lobe of the lung, then to the lower part of the lung adjacent to the diaphragm, then to the liver, and finally to the abdomen in the region of the large intestines; frequently returning over the same track. It is not uncommon for this moving, jumping pain to lessen in intensity in one organ or part of the body, and to increase in a corresponding ratio in another organ at a considerable distance. I have seen the left eye of a patient contracted or shrunken and turned in toward its inner canthus, and vision so impaired that objects appeared double, the pain being much more acute in the region of the lungs and liver than in the head. In two instances I have seen numbness of the extremities; in all there was venous congestion and generally a corresponding increase of action in the arteries. In one of the ten cases hereafter spoken of, there was both venous and arterial congestion, and the pulse never rose to the healthy standard, either in volume or stroke, until the patient, a negro woman, became convalescent; and even after she began to expectorate freely, the oscillation of the artery at the wrist was not perceptible. The Cold Plague assumes many different garbs in which to make its appearance, one of which, as already remarked, is no doubt the Black Tongue; I have known abscesses to form in the early stages, in two instances in the soft parts of the lower jaw; in another case the lips were excoriated and much swollen. I consider that the lungs are primarily affected, the liver secondarily; the liver is engorged, though the engorgement may only produce a suspension of the secretory process, sometimes perhaps causing irritation or inflammation to supervene.

The Malignant Billious Pneumonia in its metastatic state yields to stimulants and diaphoretic ptisans. Brandy and water I consider an excellent remedy in the incipient stages, during the metastasis of the pain, and I gave it in several instances with desirable effect. A tea made of the Eupatorium perfoliatum, sufficiently strong to act as an emetic or purgative for a short time, then diluted and given as a diaphoretic, is also a valuable remedy in the first stages of the affection. But whenever the disease becomes seated on any organ of the body, the antiphlogistic treatment seems generally to have the best effect; and in no case where expectoration had not taken place, did I dare to give stimulants, opiates, or animal diet in any form. I have found farinaceous diet to be the most suitable, especially before expectoration takes place. Before expectoration has become free, the principal articles of diet I use are rice water, corn gruel, rice, grits, and the crust of corn bread browned by the fire.

I had ten cases of the Cold Plague on a single plantation in Forsyth county, situated on the Chattahoochee river, a rapid mountain stream. The place had been settled about fifteen years, during which time, according to the positive declarations of the occupants, no endemic epidemic, or disease of any kind, had affected those that lived upon it. The appearance of so many cases upon one farm cannot therefore be attributed to any local causes. Most of the persons that visited the plantation during the prevalence of sickness upon it were more or less affected; while none of the neighbors that did not visit the sick were molested by the malady. While in attendance, I was myself twice attacked with it, the first time lightly, the second more severely.

Case 1. Miss E. B. aged 16. This is the case mentioned in which an eye was affected. Symptoms presented themselves in the following order: rigors, general coldness of the body and extremities, sore throat, slight pain in the side, and in the diaphragmatic region of the right lung and liver. The arterial action was considerably increased, with evident venous congestion. A vein was opened in the arm and but little blood taken, which indicated congestion in the superficial veins at least; she began to prostrate, and the artery at the wrist to thread, the bleeding was stopped, and I have not used the lancet in any case since. The tongue thickened and enlarged laterally, as the irritation or inflammation of the lungs and liver increased; it also became coated with a brown fur, and was red at the tip and edges; the skin was dry, the face flushed, with slightly impeded respiration, and considerable nervous excitement, but not so as to affect the mind. The stomach was in an abnormal state, nauseants in the form of tartar emetic water and ipecac. producing no more effect upon it than so much cold water. I gave her a combination of calomel, nitrate potass, and tartar emetic, in the proportion of calomel 2 gr., tartar emetic $\frac{1}{4}$ gr., nitrate potass 2 gr., every two hours, and put her in a warm saline bath whenever she would bear it; the skin continued dry, and the medicine produced no effect. I now gave her, in connection with the use of the bath, strong tea of the Eupatoriam perfoliatum; still the medicine and remedies externally had no evident action, save to lessen the pain in the side from the application of a heated dough cake that I had directed to be used in the intervals of bathing. I told the patient that if she did not expectorate soon she would die. At the desire of her father, another physician was called in to consult; we determined to apply blister plasters to the inferior extremities and sinapisms to the superior, and to the diaphragmatic region of the lung and liver of the right side, continuing the use of the bath. No change was produced for the better. A saline enema was ordered, which resulted in a watery evacuation, indicating a suspension of secretion from the liver. The case was again placed under my sole care.

It occurred to me that the application of a sinapism to the epigastric region would rouse the dormant nerves of the stomach, and I was gratified to find after the application that that organ responded to the remedies used. A table spoonful of tartar emetic water, (tar. em. grs. vi., water ℥iv.,) was given every fifteen minutes for a short time; it lessened the febrile excitement a little, but did not produce expectoration. A mixture of oxymel of squills and Coxe's hive syrup was used next, till it produced an emetic effect—the lady ejected from the larynx and bronchial tubes, a handful of vitiated mucous, a great part of which was of the consistence of old cheese, and of a purulent cast. The consulting physician, upon first examination, thought the principal seat of the disease in the liver, but when I directed his attention to the mucous râle in the throat and bronchial tubes, it produced a change in his views. The diet was strictly vegetable and consequently antiphlogistic, except during the brief period of the consultation. The consulting physician wished that chicken water with red pepper should be given, to which I objected, signifying that the inflammation was too high in the lungs, and that there might be gastritis, as the point of the tongue was red, though there was no tenderness on pressure in the epigastric region. I agreed to make trial of plain chicken water, a table spoonful every three or four hours; but it increased the febrile excitement, and was abandoned.

An expectorant of oxymel of squills and hive syrup, with a diaphoretic ptisan of Eupatorium perfoliatum and horehound, with an occasional pill of blue mass, were the chief remedies used during her convalescence.

*Case* 2. Ann B., a little girl aged 4 years; had two attacks, both ushered in by the same general coldness and rigors, with pain in the frontal portion of the head. She appeared to have recovered from the first, which was a light attack, and to have been in good health for about three weeks, when symptoms of a return of the disease made their appearance, followed by spasms and signs of apoplexy. The warm saline bath, cold applications to the head, and sinapisms to the extremities, removed the apoplexy, and restored the circulation of the fluids nearly to their healthy action. The tongue thickened and enlarged transversely during the high inflammatory excitement. The mucous tissue of the throat was irritated or inflamed, as has been the case with all the other cases. Thinking that the tendency to apoplexy was produced by worms, I directed a vermifuge to be given, followed by a mercurial purgative; it caused the expulsion of many ascarides. There was considerable pain in the diaphragmatic region of the right lung and liver. The application of the heated dough cake to the side, the use of nauseants, diaphoretics, expectorants, and the occasional use of the blue pill, with diet similar to that in case 1, soon removed the complaint.

*Case* 3. C. B., a young man 19 years of age, was taken with coldness and rigors, sore throat and pain in the frontal region of the head; tongue pale, with a scarcely perceptible thin coat of white fur, and pain in the diaphragmatic region of the lungs and liver. As the congestion, irritation and inflammation increased in the liver and lungs, the tongue became swollen, the fur became thick, yellowish, and finally brown. Although this was a severe case, I had no difficulty with it, as the stomach and general system responded to the remedies given. As soon as expectoration became free and continued, the disease subsided.

*Case* 4. Eliza, a negress, aged 25; had the usual precursors, general coldness, and rigors, sore throat, pain in the frontal part of the head, in the diaphragmatic region of the lungs and liver, pain in the renal region of the back, with apparent congestion in the superficial veins and arteries. There was also general prostration and relaxation that seemed to affect the superficial vessels of the body, numbness of the arms and hands; tongue pale, with a very thin coat of white fur. This is the case referred to in which the pulse did not rise to the healthy standard till convalescence was nearly perfected. The system responded to the remedies taken internally, nauseants produced almost immediate secretion from the lungs, and as expectoration increased she improved, and under the application of sinapisms to the extremities, the heated dough cake to the side, a stimulating liniment to the back, and a tepid saline bath to the feet, with a continued use of nauseants, diaphoretics, ptisans, a mercurial whenever the bilious secretion appeared to be impeded, and expectorants, she recovered. A sinapism applied to the spinal column commencing at the inferior part of the neck and ending at the middle of the back, soon removed the numbness from the arms and hands.

*Case* 5. Columbus, a negro boy, four years old; indicating symptoms, sore throat, general coldness and rigors, pain in front part of the head, in the diaphragmatic region of the lungs and liver, in the abdominal region, apparently in the large intestines. The tongue was covered with a thick coat of white fur, and as the disease advanced, this organ enlarged and reddened at the sides and point, the fur assuming a brownish cast. The arterial action was much increased, with evident superficial venous congestion. The warm saline bath was used, cold applications to the head. An aqueous solution of sulphate magnesia and tartar emetic (℥i. of salts, ℥iv. of water, 1 gr. tartar emetic) was administered in doses of one teaspoonful every fifteen miuutes, until it acted on the bowels three times. The lips commenced healing under this treatment, and with the use of the Eupatorium perfoliatum as a diaphoretic, and the oxymel of squills with hive syrup as an expectorant, and an occasional blue pill to stimulate the liver to proper action, the patient recovered. The diet was the same as in the other cases.

The cause, whatever it be, of malignant bilious pneumonia, I conceive to be inhaled into the mouth and nostrils, and by producing its action on the Schneiderian tissue of the nose, and the mucous membrane of the mouth and fauces, it creates the cold plague. That the mucous tissue, pleura and parenchyma of the lungs are involved in this disease, I think no one will deny.

